# Single Stage Repair of #30 Facial Cleft with Bone Morphogenic Protein

**DOI:** 10.1097/GOX.0000000000001937

**Published:** 2018-11-13

**Authors:** Clifford C. Sheckter, Paul Mittermiller, Kay Hung, Zeshaan Maan, Danielle Rochlin, Robert M. Menard

**Affiliations:** From the * Division of Plastic & Reconstructive Surgery, Stanford University. Stanford, CA; † Department of Plastic Surgery, Northern California Kaiser Permanente Craniofacial Clinic, Kaiser Permanente Santa Clara, Santa Clara, Calif.

## Abstract

Tessier #30 clefts (median mandibular clefts) represent a spectrum of deformities ranging from a minor cleft in the lower lip to complete clefts of the mandible involving the tongue, lower lip, hyoid bone, thyroid cartilages, and manubrium. Various techniques have been used to address these problems; the most common procedure involving 2 stages: an initial correction of the soft tissue followed by closure of the mandibular cleft at a later date using bone grafting. This approach was subsequently reduced to a single operation, but still required harvesting of autologous bone graft. Here, we describe a modified single-stage operation using human recombinant bone morphogenic protein, avoiding bone graft harvest and allowing for simultaneous treatment of bone and soft tissue.

## INTRODUCTION

Median clefts of the mandible, also known as Tessier #30 facial clefts, are a rare congenital anomaly that was first reported in 1819 by Couronné according to Monroe.^1^ To date, fewer than 100 cases have been described in the literature^2^ and treatment often occurs in 2–3 stages.^3^ #30 clefts vary in severity of presentation, ranging from a minor notch in the lower lip to complete clefts of the mandible involving the tongue, lower lip, hyoid bone, thyroid cartilages, and manubrium. Collectively these defects are both functionally and cosmetic problematic.^3–5^ The management of #30 clefts has evolved over the years. Initially, the deformity was treated in 2 stages mirroring the common treatment paradigm of palatal clefts,^3,6,7^ with initial correction of the soft tissue followed by bone grafting of the mandibular cleft at a later date.^8^ This approach was subsequently described as a single-stage procedure,^9^ yet still required 2 surgical sites and the morbidity of bone graft harvest, typically from rib or iliac crest.^3,9^

Bone morphogenic proteins (BMPs) are multifunctional growth factors that are approved for use in spinal fusion^10^ with off-label uses in treatment of long bone defects and nonunions and fracture repair.^11^ Their use has also been described in the maxilla for cleft palates.^12^ Here, we describe the use of BMP in successful single-stage treatment of a #30 facial cleft without need for bone graft harvest.

## METHODS AND SURGICAL TECHNIQUE

**Fig. 1.**
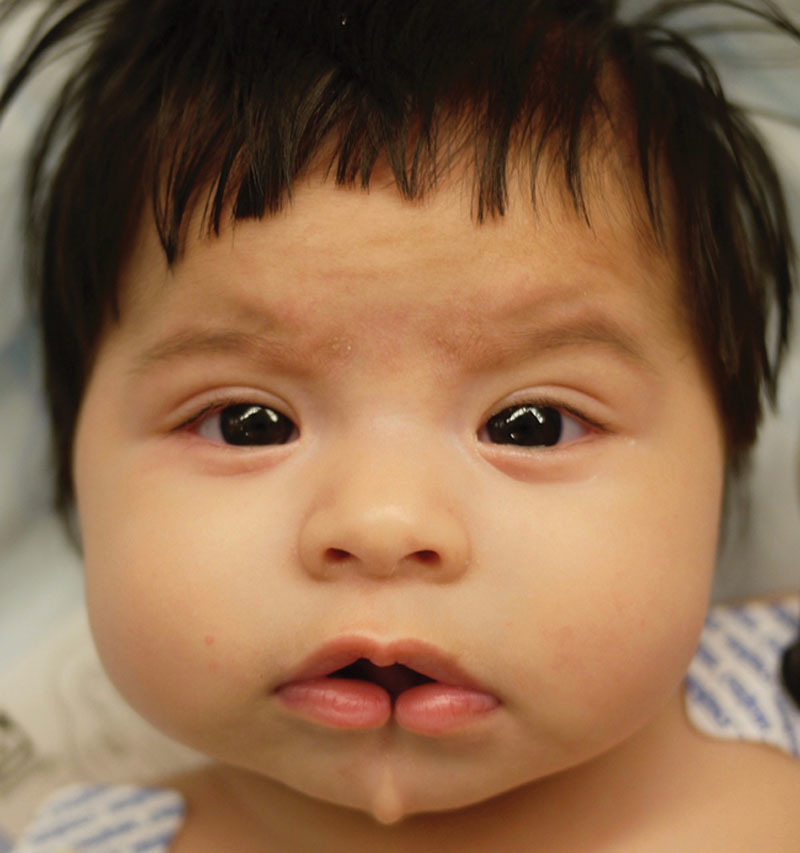
Preoperative photograph demonstrating soft-tissue median mandibular cleft with aberrancies at lip and chin.

**Fig. 2.**
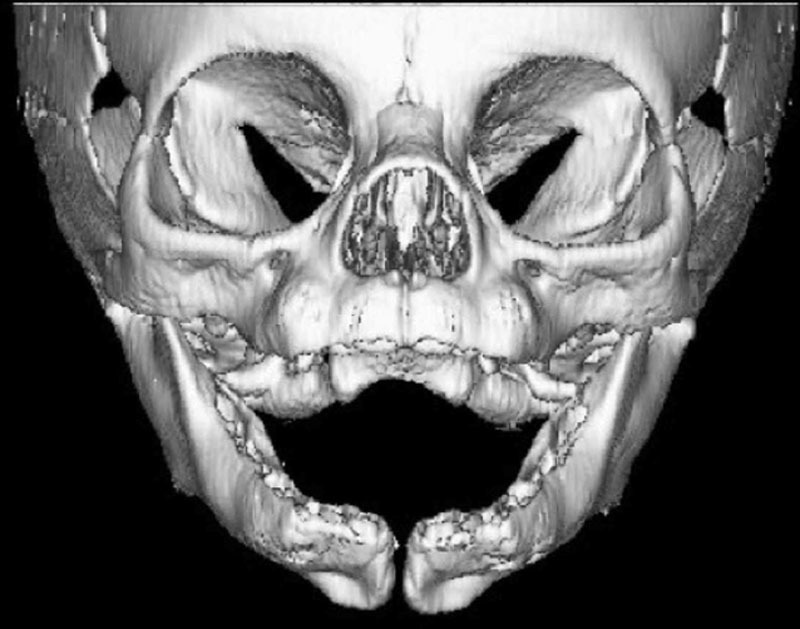
Preoperative computed tomography scan of face demonstrating median mandible defect.

The Kaiser Permanente Institutional Review Board approved the following exhibition. Patient consent was given. Computed tomography scans with 3-dimensional reconstruction were obtained preoperatively to delineate the bony defect (Fig. 1). The child was 4.5 months old at the time of surgery. The extent of external defect was marked using loupe magnificent to avoid discarding healthy tissue (Fig. 2). The margins were sharply incised, and dissection was carried out down to the level of the mandibular diastasis. Bony margins were well identified, including aberrant connective tissue in the mandibular gap, which was excised with needlepoint cautery.

A 5-mm bony gap was cleanly visualized and reduced to a 3-mm gap after burring small holes on either side and cinching with 26-gauge wire. Using a 3-mm burr, periosteum was removed from the mandibular margins in preparation for the graft. A 0.7 mL aliquot of Infuse human recombinant bone morphogenic protein (rhBMP) (Medtronic, Dublin, Ireland) was prepared according to manufacturer’s instructions and placed in the mandibular gap. A 1.5 mm five-hole LactoSorb (Zimmer Biomet, Warsaw, Indiana) plate was fashioned along the inferior border of the mandible to avoid dental root injury. The midline hole was centered in the gap, and the 2 lateral holes were anchored using self-tapping 5-mm screws. Following secure placement, the superior cinch wire was removed.

After appropriate boney fixation, the soft tissues were addressed. The lingual frenulum was released 2 cm toward the tongue base. At the lip, the inferior orbicularis was delicately delineated and approximated with absorbable monofilament suture. All mucosal tissues were then approximated with alternating interrupted resorbable braided and gut sutures. The external lip was closed linearly in 2 layers with absorbable monofilament suture, and the vermillion was precisely approximated with fast-absorbing gut suture. A film of surgical glue was placed on the external lip skin.

**Fig. 3.**
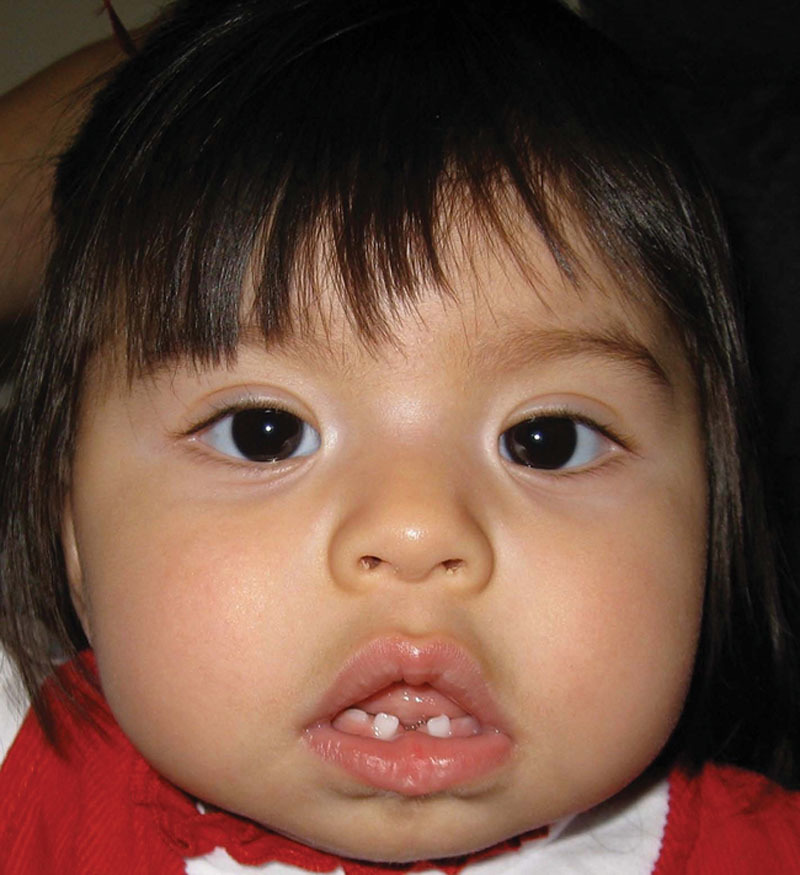
Postoperative photograph demonstrating well-healed soft tissues of lower lip and chin.

**Fig. 4.**
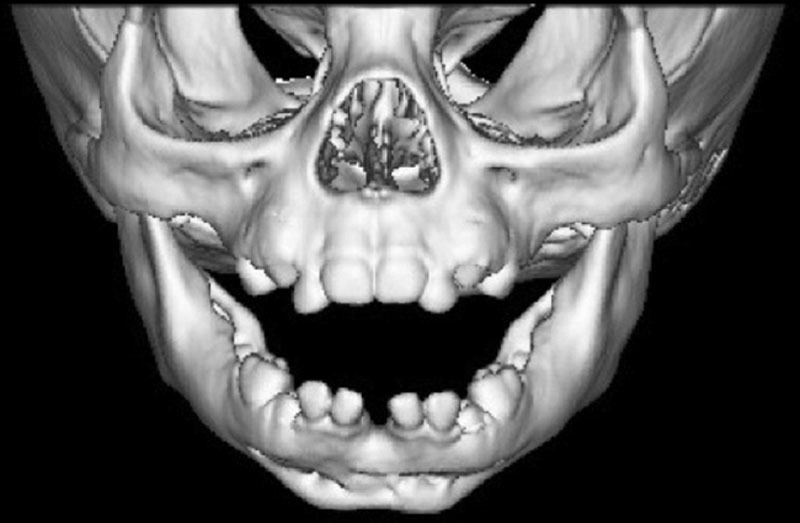
Postoperative computed tomography scan of face demonstrating union at mandible symphysis.

The child healed uneventfully. Computed tomography imaging 6 months postoperatively confirmed bony union and a well-healed scar (Figs. 3, 4). He has normal occlusion and mandibular function at 4-year follow-up. The gap between the lower medial incisors will be addressed with orthodontics in early adolescence.

## DISCUSSION

rhBMP has been successful in treating alveolar clefts^12^ and performed equally well in treating this mandibular cleft. The benefits of rhBMP in the treatment of #30 facial cleft include (1) completion of the repair in a single operation; and (2) elimination of the need bone graft harvest and the associated morbidity. The drawbacks of rhBMP include cost and the potential for malignancy, though this has only been observed in cases of spine fusion^10^ and has not been described in craniofacial applications. Though rhBMP is an expensive material that adds direct cost to the treatment, this must be weighed against costs that can be attributed to bone graft harvest, including additional operating room time, additional hospitalization for pain control, and the costs of a second surgery if the staged approach is selected. Of note, rhBMP is not labeled for this use by the Food and Drug Administration, and thus this technique is experimental. In smaller bony defects without significant gapping, it may be feasible to close periosteum without rhBMP and achieve union, although these authors have not encountered such a case.

The strength of union appears as strong as bone grafting, given the child has normal mandible function on long-term follow-up. This bone defect size varies within #30 facial clefts, and clearly this technique was satisfactory for a 5-mm defect. It is possible that larger defects may not be amendable to this technique. The scarring to lower lip is minimal at 4-year follow-up. Additional considerations for scar reduction can include incorporating a z-plasty just under the white roll at the time of primary repair.

In summary, our report suggests that #30 clefts can be safely and effectively treated with BMP in a single stage and should be considered as a potential option in other craniofacial bony defects. Further studies, including comparative and cost-effectiveness analyses, are needed to clarify the routine role of rhBMP in the management of craniofacial bony defects.
